# Knockdown of TRPV Genes Affects the Locomotion and Feeding Behavior of *Nilaparvata lugens* (Hemiptera: Delphacidae)

**DOI:** 10.1093/jisesa/ieaa002

**Published:** 2020-02-15

**Authors:** Jinghua Zhu, Xiaoqing Liu, Kunmiao Zhu, Hanyu Zhou, Liang Li, Zengxin Li, Weiwei Qin, Yueping He

**Affiliations:** Hubei Insect Resources Utilization and Sustainable Pest Management Key Laboratory, College of Plant Science and Technology, Huazhong Agricultural University, Wuhan, China

**Keywords:** transient receptor potential, *Nilaparvata lugens*, RNAi, feeding behavior, electropenetrography

## Abstract

The vanilloid-type transient receptor potential (TRPV) channel is reported to be the molecular target of the commercial insecticide pymetrozine, which specifically disrupts the feeding of plant sap-sucking insects. However, the functions of TRPV channels in plant sap-sucking insects have not been fully elucidated. In the present study, RNA interference was used to investigate the effects of the knockdown of TRPV genes (*Nan* and *Iav*) on the mortality, locomotion, and feeding behavior of an important plant-feeding insect pest in rice, the brown planthopper, *Nilaparvata lugens*. Injecting dsRNA of *Nan* and *Iav* into fourth-instar nymphs significantly knocked down the target genes. The injection of ds*Nan* or ds*Iav* did not affect any morphological phenotype (including leg extension) of *N. lugens* nymphs and adults. Knockdown of *Nan* or *Iav* resulted in significantly decreased climbing activity against top plants but did not influence the leg-griping strength of adults. Knockdown of *Nan* resulted in a significantly elevated mortality of *N. lugens* in the observation period of 7 d after injection, whereas no significant difference in survival rates 7 d after injection was found between ds*Iav*-injected and ds*GFP*-injected insects. Electropenetrographic (EPG) recordings indicated that knockdown of *Nan* and *Iav* reduced the ingestion activity in the rice phloem tissues of *N. lugens*. Knockdown of *Nan* and *Iav* significantly reduced the amount of honeydew excreted by *N. lugens*. Our findings indicated a relationship between TRPV and *N. lugens* locomotion and feeding behavior, which may help to fully elucidate the functions of TRPV in insects.

Recent research discovered a unique, novel insecticide target, the vanilloid-type transient receptor potential (TRPV) channel, which is the binding target of three insecticides, pymetrozine, pyrifluquinazon, and afidopyropen (Nesterov et al. 2015, Kandasamy et al. 2017, Wang et al. 2019). Nesterov et al. (2015) showed that pymetrozine and pyrifluquinazon activate the *Drosophila* TRPV channel complex, encoded by *Nanchung* (*Nan*) and *Inactive* (*Iav*) genes, and silence antennal chordotonal stretch receptor organs that are essential for hearing and gravity sensation. It was found that pymetrozine can also activate Nan-Iav channels of plant sap-sucking insects, *Acyrthosiphon pisum* (Hemiptera: Aphidoidea) and *Nilaparvata lugens* based on an in vitro cell assay (Kandasamy et al. 2017, Wang et al. 2019). These insecticides were thought to selectively block feeding of plant-sap sucking insects, such as aphids, whiteflies, and planthoppers (Harrewijn and Kayser 1997, Fuog et al. 1998, He et al. 2011a, Raj Boina et al. 2011, Kang et al. 2012, Leichter et al. 2013, Maienfisch 2019). However, there are few reports describing the functions of TRPV genes in plant sap-sucking insects. In this study, the effects of knockdown of *N. lugens* TRPV genes on mortality, locomotion, and feeding behavior were investigated.

The brown planthopper, *N. lugens*, is an important rice pest in Asian countries. *N. lugens* causes severe yield reduction of rice and significant economic loss due to its ingestion of rice plant sap and transmission of plant viruses (Sōgawa 1982). Chemical control is a common method to manage *N. lugens* populations in China and other Asian countries. Since 2005, pymetrozine has been widely used for *N. lugens* control in China. Recently, it was reported that field populations of *N. lugens* have developed high-level resistance to pymetrozine (Wu et al. 2018). Therefore, there is an urgent need to understand the toxicity mechanism of pymetrozine to *N. lugens* and the resistance mechanism of the insect to pymetrozine.

## Materials and Methods

### Insect

The tested planthoppers were collected in paddy fields in Huazhong Agricultural University, Wuhan, China, and were reared continuously on rice seedlings of the Taichung Native l (TN1) variety in the laboratory at 28 ± 0.5°C, 70% ± 10 % humidity, and a photoperiod of 14:10 (L: D) h.

### RNA Extraction and cDNA Synthesis

Total RNA was isolated from the heads of *N. lugens* adults using TRIzol Reagent (Invitrogen, Carlsbad, CA) according to the manufacturer’s protocol. RNA purity was checked using a NanoPhotometer spectrophotometer (IMPLEN, Westlake Village, CA). First-strand cDNA was synthesized from 2-μg total RNA using *TransScript* One-Step gDNA Removal and cDNA Synthesis SuperMix (Transgen, China) according to the manufacturer’s instructions.

### RNA Interference

The cDNA fragments of *N. lugens Nan* (KX249697), *N. lugens Iav* (KX249698), and the green fluorescent protein (GFP) gene were amplified by polymerase chain reaction (PCR) using primers containing the T7 RNApolymerase promoter (Supp Table 1 [online only]). The products were gel purified and used as templates to synthesize dsRNA, using the MEGAscript T7 High Yield Transcription kit (Thermo Fisher Scientific, Waltham, MA). The resulting dsRNAs were dissolved in ultrapure water, and the quality and concentration were determined by agarose gel electrophoresis and a Nanodrop 2000 spectrophotometer. DsRNA injection was performed on fourth-instar nymphs of *N. lugens*. Specifically, 150 ng of dsRNA (5 μg/µl) of *Nan*, *Iav*, or GFP was injected into the junction of the prothorax and mesothorax of each fourth-instar nymph following the protocol described by Liu et al. (2015). Surviving numbers and morphology of tested insects on TN1 rice seedlings were recorded at 24-h intervals for 7 d. The treated nymphs were first placed in Petri dishes with moist TN1 rice seedlings for 1 d of recovery, and 15 healthy nymphs were subsequently transferred into a plastic cup (3-cm bottom diameter × 25-cm length) with 10 TN1 rice seedlings. Each treatment was repeated three times. Petri dishes, plastic cups, and feeding chambers with treated nymphs were placed at 26 ± 1°C, 70% ± 10 % RH, and a photoperiod 14: 10 (L: D) h.

### Analysis of RNAi Efficiency

The interference efficiency in the entire body was checked using quantitative real-time PCR (qRT-PCR): five insects that were selected randomly at the seventh day after injection of dsRNA for subsequent RNA extraction and cDNA synthesis. Three biological replicates were conducted in each treatment. The qRT-PCR assay was performed on an ABI Prism 7300 (Applied Biosystems, Foster City, CA) using SYBR Premix Ex Taq (Takara Biotechnology Corporation Co. Ltd, Dalian, China). *Nlactin1* (GenBank EU179846.1) was used as an internal standard to normalize cDNA concentrations. The primers for qRT-PCR are presented in Supp Table 1 (online only). Quantitative reactions were performed on three technical replicates. The expression level of the target gene was normalized using the 2^−ΔΔCt^ method (Livak and Schmittgen 2001).

### Morphology Observation and Behavioral Assays

The morphological phenotypes of *N. lugens* nymphs and adults after the dsRNA injection were examined. The images of 30 insects 7 d after dsRNA injection were recorded and the angle of hindleg femur-tibia joint was measured by Photoshop CS 8.0.1 version (Adobe Systems, Inc, San Jose, CA) based on each image.

Climbing assays were conducted by the modified method according to the method reported by Nesterov et al. (2015). Ten treated adults (6–8 d after dsRNA injection) were placed on the bottom of a glass vial (height: 130 mm). Then, four plant seedlings with the whole roots covered by wet cotton were inverted into the top of one glass vial. For each treatment, two biological replicates and each biological replicate consisted of three technique replicates with 10 individuals in each vial were performed. Climbing scores were determined at 1–9 h by counting the insects in the upper half of the vial; data are presented as percentages.

Gripping assays were carried out using the same vials as climbing assays. Four plant seedlings with the whole roots covered by wet cotton were placed on the bottom of a glass vial. More than 10 treated adults (6–8 d after dsRNA injection) were released close to the plants. When 10 adults had climbed on the plants, the vial was slowly inverted. Insects that dropped from the plants were counted immediately. Two biological replicates and each biological replicate consisted of three technique replicates were conducted in each treatment. Gripping scores were calculated as the percentages of the insects gripping onto the plants.

### Feeding Behavior and Honeydew Excretion

To measure the effect of knockdown of *Nan* and *Iav* on feeding of *N. lugens*, DC electropenetrography (EPG) was used to monitor feeding behavior of individual dsRNA-treated female adult on a susceptible TN1 rice plant. EPG was recorded in a Faraday cage using a Giga-4 DC EPG system with a 10^9^ Ω input resistance and an input bias current of <1 pA (Wageningen Agricultural University, Wageningen, the Netherlands). One TN1 rice plant in the tillering stage was placed in a glass tube, with the whole root being dipped in water, within which a plant electrode of a copper wire (2-mm diameter × 10-cm length) was inserted. One *N. lugens* female, which had emerged after 2–4 d (6–8 d after dsRNA injection) was connected to an insect electrode via the thoracic notum using a gold wire (Ø 18 µm, Wageningen Agricultural University) and a silver conductive glue (Wageningen Agricultural University) and was then carefully placed onto the plant stem. All EPG tests were conducted at 26 ± 1°C and 70 ± 10 % RH under continuous light conditions for 6 h. Eighteen replicates were recorded for each treatment and used for final data analysis.

The EPG signals were analyzed using PROBE 3.0 software (Wageningen Agricultural University). The EPG waveforms from recordings on plants were classified into seven types according to the categories: np for nonpenetration, Nc (N1 + N2 + N3) for pathway phase (including penetration initiation (N1), stylet movement and salivation (N2), and extracellular activity near the phloem region [N3, also known as the X wave]), N4 (N4-a + N4-b) for stylets in the phloem tissue (including an intracellular activity [N4-a], and sustained phloem sap ingestion [N4-b] in the phloem tissue) termed phloem ingestion phase, and N5 for stylets in the xylem tissue termed putative xylem ingestion phase (Seo et al. 2009, He et al. 2011a). EPG waveform variables (including nonsequential and sequential variables) were analyzed using the same method as reported by He et al. (2011a). Two nonsequential response variables were calculated similarly to those in Backus et al. (2007). The number of waveform events per insect (NWEI) was the number of times that a given waveform appeared during the recording time per individual. The waveform duration per insect (WDI) was the sum of the durations of all events of a given waveform per individual. Mean number of probes per insect was the mean value of the total number of probes made by each insect during the recording time. Two sequential variables calculated were the duration from the start of the experiment to the first probe (N1) and the duration of the first probe.

Additionally, the amount of honeydew, an indicator of the amount of food intake, excreted from a treated female adult (6–8 d after dsRNA injection) was measured using a parafilm sachet (4 × 4 cm) positioned on a healthy stem of TN1 rice plant (Pathak et al., 1982), after feeding on a TN1 plant for 24 h. The experiment was replicated 15 times.

### Statistical Analysis

Real-time qPCR results were analyzed by analysis of variance followed by a multiple comparison of means (LSMEANS with Tukey–Kramer multiple comparison tests) using Proc GLM procedures of SAS software (version 8.01, SAS Institute Inc.). Insect survival curves were made with the Kaplan–Meier method and comparatively analyzed with a log-rank (Mantel-Cox) test using GraphPad Prism software (version 7.0; GraphPad Inc.). The data of climbing assay and gripping assay were analyzed by Mann–Whitney *U*-test. The femur-tibia angle data, honeydew data, and EPG data between different treatments were compared by SAS Proc GLM procedures. The significance level was set to *P*  <  0.05.

## Results

### High Mortality After Suppressing *Nan* Rather Than *Iav*

**Fig. 1.**
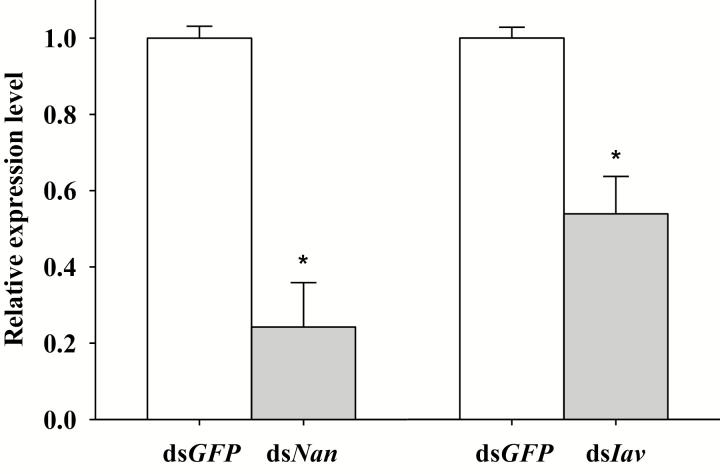
Mean transcript levels in whole bodies of *Nilaparvata lugens* at 7 d after injection with dsRNA of *Nan*, *Iav* or *GFP*. *Indicates a significant difference (*P* < 0.05) between ds*GFP* and ds*Nan* or ds*Iav* treatments. Data were expressed as the mean ± SEM.

The expression of *Nan* and *Iav* genes was effectively knocked down using RNAi technology. The results showed that after dsRNA injection of *Nan* into fourth-instar nymphs, the transcript level of *Nan* 7 d after injection was reduced by 75.8% ± 11.6% (*F*_1,5_ = 28.34, *P* = 0.006; Fig. 1) compared with the control in which ds*GFP* was injected. The injection of *Iav* dsRNAs into fourth-instar nymphs resulted in an 46.1 ± 9.8% decrease in transcript levels (*F*_1,5_ = 14.78, *P* = 0.018; Fig. 1) compared with the ds*GFP-*injected group.

**Fig. 2.**
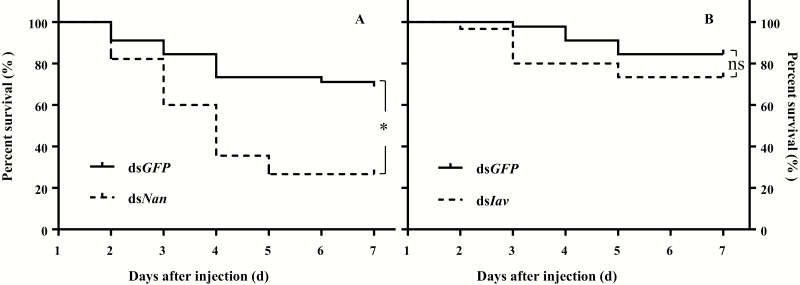
Survival curves of *N. lugens* 1–7 d after injection with dsRNA of *Nan*, *Iav* or *GFP*, when fed on rice (A and B). (A) A log-rank (Mantel-Cox) test using GraphPad Prism software showed a significant difference (*) in survival rate between ds*GFP* and ds*Nan* treatments (*P* < 0.05). (B) A log-rank (Mantel-Cox) test showed no significant difference (ns) in survival rate between ds*GFP* and ds*Iav* treatments (*P* > 0.05).

Survival curves demonstrated that knockdown of *Nan* resulted in significantly decreased survival of *N. lugens* (χ ^2^ = 15.93, *P* < 0.0001; Fig. 2A), while suppressing the expression level of *Iav* did not cause significantly different survival of *N. lugens* (χ ^2^ = 2.09, *P* = 0.15; Fig. 2B). After 5 d of injection of ds*Nan* into fourth-instar nymphs, only 26.67 ± 6.67% of insects survived, which is significantly lower than the survival rate of 73.33 ± 3.85% observed in the ds*GFP*-injected group (*F*_1,5_ = 36.75, *P* = 0.0037; Fig. 2A).

### Knockdown of *Nan* and *Iav* Genes Affects the Climbing Behavior of *N. lugens*

The injection of ds*Nan* and ds*Iav* did not influence any morphological phenotype of *N. lugens* nymphs or adults (Supp Fig. 1 [online only]). No significant difference in the angle of hindleg femur-tibia joint was found between ds*GFP*-injected group and ds*Nan*- or ds*Iav*-injected group (*F*_2,179_ = 1.81, *P* = 0.166; Supp Fig. 1 [online only]).

**Fig. 3.**
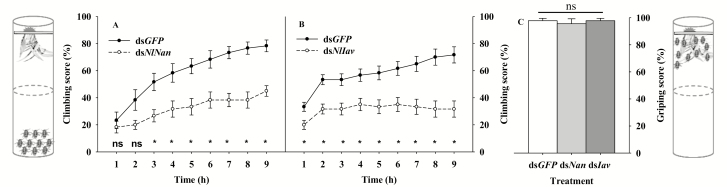
Climbing assay (A and B) and gripping assay (C) for *N. lugens* adults after being injected with dsRNA of *Nan*, *Iav* or *GFP*. *Indicates a significant difference (*P* < 0.05) between ds*GFP* and ds*Nan* or ds*Iav* treatments. ‘ns’ indicates no significant difference (*P* > 0.05) between ds*GFP* and ds*Nan* or ds*Iav* treatments. Data were expressed as the mean ± SEM.

Climbing assay demonstrated that knockdown of *Nan* or *Iav* resulted in significantly decreased climbing activity from the tube bottom up rice plants that were inverted on the tube top (*P* < 0.05; Fig. 3A and B). The climbing score was no more than 50% during 9 h in the ds*Nan*- or ds*Iav*-injected group, whereas higher than 70% after 8h in the ds*GFP*-injected group. However, knockdown of *Nan* or *Iav* did not influence the leg-griping strength of adults (Fig. 3C). Almost 100% of adults from ds*GFP*-, ds*Nan*-, or ds*Iav*-injected group can grip the plants and not drop down, after the tube was gently inverted (χ ^2^_2,17_ = 0.88, *P* = 0.6432; Fig. 3C).

### Knockdown of *Nan* and *Iav* Genes Affects the Feeding Ingestion and Honeydew Excretion of *N. lugens*

**Fig. 4.**
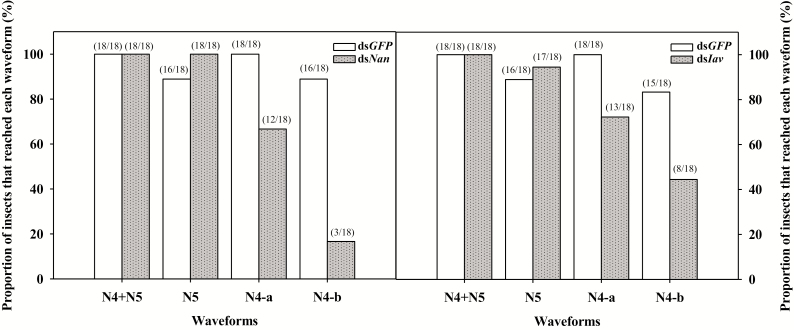
Proportion of adults that reached each EPG waveform during feeding on a rice plant after RNAi. N4 (N4-a + N4-b) for stylets in the phloem tissue (including an intracellular activity (N4-a) and sustained phloem sap ingestion (N4-b) in the phloem tissue) termed phloem ingestion phase; N5 for stylets in the xylem tissue. The number in the parentheses upon each column means the number of the insects reached N4, N4-a, N4-b, or N5 waveform/all the recording replicates.

After suppressing the expression level of *Nan* and *Iav,* the activities in the phloem ingestion phase of *N. lugens* female adults were significantly inhibited when fed TN1 rice plants (Fig. 4; Tables 1 and 2). All ds*GFP*-injected individuals reached the phloem tissues (N4-a and Na-b), whereas approximately 30% of ds*Nan-* or ds*Iav*-injected adults did not (Fig. 4). The ds*Nan-* or ds*Iav*-injected individuals that reached the phloem still spent significantly less time in phloem phase than did the ds*GFP*-injected insects (Tables 1 and 2). During the 6-h recording period, the mean waveform duration per insect (WDI) for ds*Nan*-injected individuals was significantly lower than (only 23.6% of) that in the ds*GFP*-injected group (*F*_1,29_ = 2.04, *P* < 0.001; Table 1). The ds*Nan*-injected individuals also performed significantly fewer events (NWEI) in phloem tissues than did the ds*GFP*-injected insects during the 6-h recording period (*F*_1,29_ = 0.81, *P* = 0.023; Table 1). In contrast, the ds*Nan-*injected individuals performed more activities in xylem tissues: all ds*Nan*-injected individuals reached the xylem tissues (Fig. 4), performed more events in xylem issues (*F*_1,33_ = 4.56, *P* = 0.008; Table 1), and spent significantly more time ingesting xylem sap than did the ds*GFP*-injected insects (*F*_1,33_ = 11.2, *P* = 0.004; Table 1). Moreover, WDI values for the nonpenetration phase and pathway waveforms were significantly increased after ds*Nan* injection (*P* < 0.05; Table 1).

**Table 1.** Comparison of 6-h EPG response variables of *Nilaparvata lugens* feeding rice plants after injected with ds*GFP* and ds*Nan*

Variables	ds*GFP*	ds*Nan*	*F* (df), *P*
1. Mean time to first probe (min) per insect	5.5 ± 2.1 (18)	5.8 ± 1.8 (18)	0.05 (1,35), 0.915
2. Mean duration of first probe (min) per insect	48.5 ± 26.8 (18)	28.7 ± 9.2 (18)	3.44 (1,35), 0.489
3. Mean number of probes per insect	6.9 ± 1.1 (18)	8.8 ± 0.8 (18)	3.89 (1,35), 0.183
4. WDI for np (min)	26.9 ± 5.5 (18)	76.5 ± 18.2 (18)*	9.63 (1,35), 0.013
5. WDI for pathway (min)	75.3 ± 13.0 (18)	114.5 ± 10.5 (18)*	0.87 (1,35), 0.024
6. NWEI for phloem phase	6.2 ± 0.8 (18)	3.4 ± 0.9 (12)*	0.81 (1,29), 0.023
7. WDI for phloem phase (min)	209.1 ± 23.7 (18)	49.4 ± 20.0 (12)*	2.04 (1,29), <0.001
8. NWEI for xylem phase	2.2 ± 0.4 (16)	5.2 ± 1.0 (18)*	4.56 (1,33), 0.008
9. WDI for xylem phase (min)	48.4 ± 11.2 (16)	119.1 ± 20.2 (18)*	11.2 (1,33), 0.004

*Indicates a significant difference (*P* < 0.05) between ds*GFP* and ds*Nan* o treatments within the same variable. Data were presented as mean ± SEM (*n*).

^1^WDI means waveform duration per insect.

^2^NWEI means number of waveform events per insect.

**Table 2.** Comparison of 6-h EPG response variables of *Nilaparvata lugens* feeding rice plants after injected with ds*GFP* and ds*Iav*

	ds*GFP*	ds*Iav*	*F* (df), *P*
1. Mean time to first probe (min) per insect	5.6 ± 2 (18)	5.3 ± 1.1 (18)	0.02 (1,35), 0.900
2. Mean duration of first probe (min) per insect	48.7 ± 26.1 (18)	34.3 ± 19.1 (18)	0.19 (1,35), 0.667
3. Mean number of probes per insect	7.7 ± 1.5 (18)	13.3 ± 2.5 (18)	3.57 (1,35), 0.068
4. WDI for np (min)	29.4 ± 6.8 (18)	40.8 ± 8.25 (18)	1.09 (1,35), 0.303
5. WDI for pathway (min)	76.8 ± 12.9 (18)	156.7 ± 20.0 (18)*	10.6 (1,35), 0.003
6. NWEI for phloem phase	6.5 ± 0.7 (18)	3.2 ± 0.6 (13)*	11.3 (1,30), 0.002
7. WDI for phloem phase (min)	209.2 ± 23 (18)	133.2 ± 27.4 (13)*	4.25 (1,30), 0.048
8. NWEI for xylem phase	2.5 ± 0.4 (17)	4.8 ± 0.6 (16)*	9.42 (1,32), 0.004
9. WDI for xylem phase (min)	49.8 ± 9.3 (17)	69.7 ± 17.8 (16)	0.89 (1,32), 0.353

*Indicates a significant difference (*P* < 0.05) between ds*GFP* and ds*Iav* treatments within the same variable. Data were presented as mean ± SEM (*n*).

^1^WDI means waveform duration per insect.

^2^NWEI means number of waveform events per insect.

In the ds*Iav*-injected group, WDI for the phloem phase was significantly less, only 63.7% of that in ds*GFP*-injected insects (*F*_1,30_ = 4.25, *P* = 0.048; Table 2). NWEI for xylem phase of ds*Iav*-injected individuals was significantly fewer than those of ds*GFP*-injected individuals (*F*_1,30_ = 11.3, *P* = 0.002; Table 2). Compared with the ds*GFP*-injected insects, the ds*Iav*-injected individuals showed a higher NWEI value for xylem phase and a higher WDI value for pathway phase (*P* < 0.05; Table 2).

**Fig. 5.**
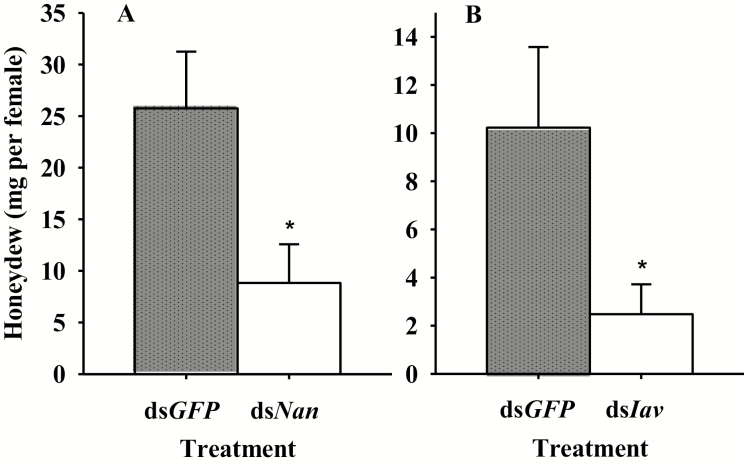
Mean amount of honeydew per day excreted by a *N. lugens* female adult after being injected with dsRNA of *Nan*, *Iav*, or *GFP*. *Indicates a significant difference (*P* < 0.05) between ds*GFP* and ds*Nan* or ds*Iav* treatments. Data were expressed as the mean ± SEM.

In *N. lugens*, the amount of food intake is directly proportional to the amount of honeydew excretion. Knockdown of *Nan* and *Iav* significantly reduced the amount of honeydew excreted by female adults of *N. lugens* after feeding on a TN1 plant for 24 h (34.0 and 24.5% of that in the ds*GFP*-injected group, respectively, *P* < 0.05; Fig. 5).

## Discussion

Pymetrozine, an insect TRPV agonist, can effectively control plant sucking insect pests. Pymetrozine-treated aphids immediately stopped feeding and eventually died from starvation (Harrewijn and Kayser 1997). Pymetrozine was slower-acting against *N. lugens* nymphs (He et al. 2011a) and was less toxic to *N. lugens* adults, but strongly suppressed the offspring numbers (Tsujimoto et al. 2015). Pymetrozine and another insect TRPV agonist, afidopyropen, can directly bind to insect *Nan* protein, rather than *Iav* protein (Kandasamy et al. 2017). In the present study, 73.3% of the ds*Nan*-injected individuals of *N. lugens* died at 5 d after dsRNA injection into fourth-instar nymphs. Current knowledge indicates that the *Nan* subunit could be a better binding target for discovery of novel insecticides for the control of plant sap-sucking insect pests.

Our previous EPG studies demonstrated that pymetrozine disturbed the feeding behavior of rice brown planthoppers and green rice leafhoppers, primarily through inhibition of phloem sap ingestion (He et al. 2011a,b). In this study, similar effects of knockdown of TRPV genes on phloem ingestion of *N. lugens* were observed. Our results suggested that there could be a relationship between TRPV and phloem feeding behavior in *N. lugens*. Additionally, knockdown of TRPV genes also affected the climbing activities of *N. lugens* against top plants. Therefore, it remains unknown whether there is direct or indirect action of TRPV on feeding of *N. lugens*. A direct effect acts on the physiological systems regulating feeding progress, while an indirect effect acts on other systems (such as locomotion behavior) causing the feeding inhibition. There are few reports indicating a direct function of TRPV on insect feeding. In *Drosophila*, *Nan* is expressed (independently of *Iav*) in labellar neurons as a mechanosensor for food hardness detection (Jeong et al. 2016). *Drosophila* flies prefer softer food at the expense of sweetness; the *Nan* mutant flies show reduced preference for softer food (Jeong et al. 2016). Notably, *Iav* mutant flies did not show a significant defect in their preference for the softer food (Jeong et al. 2016). It cannot be ruled out that TRPV directly functions on feeding behavior in plant sap-sucking insects. Similarly, it cannot be ruled out that pymetrozine may act on insect feeding systems by targeting TRPV or some additional molecular targets.

While *Nan* and *Iav* are co-expressed specifically in chordotonal stretch receptor neurons as subunits of a heteromeric TRPV channel, the individual distribution of insect TRPV is broader (Salgado 2017). For instance, *Iav* without *Nan* in *Drosophila* motor neurons regulates synaptic development and synaptic transmission (Wong et al. 2014). *Nan* without *Iav* is required in *Drosophila* to avoid dry air (Liu et al. 2007). In *N. lugens*, *Nan* and *Iav* were detected with different expression patterns in different developmental stages: *Iav* was expressed highly in the early nymphal stages, whereas *Nan* was not (Mao et al. 2018, Wang et al. 2019). In the present study, knockdown of *Nan* and *Iav* resulted in same phenotypes in morphology and locomotion but in different phenotypes in survival and feeding behaviors: knockdown of *Nan* caused a high mortality of *N. lugens* whereas *Iav* did not; the inhibition rate of the phloem phase duration in ds*Nan*-injected individuals was much higher than in ds*Iav*-injected ones. These results might indicate that *Nan* and *Iav* in *N. lugens* could play both dependent and independent roles under some conditions.

Our findings and current knowledge expand our interest in exploring the functions and applications of insect TRPV. It appears that the *Nan* protein could be more focused than the *Iav* protein for discovery of novel active compounds. However, the issue of coexpression or independent distribution of *Nan* and *Iav* in insects seems considerably complex. Whether the relationship between TRPV and *N. lugens* feeding is direct or indirect warrants further research. Phloem ingestion behavior of plant sap-sucking insects can be affected by multiple factors and regulated via different signaling pathways. The potential mechanisms of inhibition of phloem ingestion after knockdown of *N. lugens* TRPV could be: 1) a link to the chemosensory signaling system in antennae or stylets before probing or during ingestion, 2) the neuromuscular system in mouthparts that controls the movement of plant fluid and saliva, or 3) some additional signaling pathways (such as serotonin signaling pathways) related to insect feeding behavior. Future research to determine such a mechanims is warranted.

## Supplementary Material

ieaa002_suppl_Supplementary_Table_S1

ieaa002_suppl_Supplementary_Figure_S1
