# Supplementary material for: Knockdown of TRPV Genes Affects the Locomotion and Feeding Behavior of Nilaparvata lugens (Hemiptera: Delphacidae)
Source: J Insect Sci. 2020 Feb 15;20(1):9. doi: 10.1093/jisesa/ieaa002 (PMC7022682; doi:10.1093/jisesa/ieaa002)
Supplement: ieaa002_suppl_Supplementary_Table_S1 [file ieaa002_suppl_supplementary_table_s1.docx]

**Table S1 Primers used in this study**

| **Primer name** | **Primer sequence** |
| --- | --- |
| *Nan*-F | ACGGACACCTCGTCCATTAC |
| *Nan*-R | AGTGGCGTTCAACAGACAGA |
| Iav-F | ATTGGCCTGCATACCGTTCA |
| *Iav*-R | TCTTCGCCCACTGCTTCAT |
| *Nan*-qPCR-F | AAGCAACATACCAGGCATTCC |
| *Nan*-qPCR-R | GCAGTACGCTCTCTCCATAGTA |
| *Iav*-qPCR-F | GCCTCCAAATGGTGACATAAACT |
| *Iav*-qPCR-R | GCACTCCAATCTACAACTGACAA |
| *actin1*-qPCR-*F* | CCAACCGTGAGAAGATGACC |
| *actin1*-qPCR-R | GATGTCACGCACGATTTCAC |
| *Nan*-dsRNA-F | TAATACGACTCACTATAGGACGGACACCTCGTCCATTAC |
| *Nan*-dsRNA-R | TAATACGACTCACTATAGGAGTGGCGTTCAACAGACAGA |
| *Iav*-dsRNA-F | TAATACGACTCACTATAGGATTGGCCTGCATACCGTTCA |
| *Iav*-dsRNA-R | TAATACGACTCACTATAGGTCTTCGCCCACTGCTTCATC |
| *GFP*-dsRNA-F | TAATACGACTCACTATAGGGTAAACGGCCACAAGTTCAG |
| *GFP*-dsRNA-R | TAATACGACTCACTATAGGTCGGCCATGATATAGACGTT |
