# Supplementary figures and images for: Knockdown of TRPV Genes Affects the Locomotion and Feeding Behavior of Nilaparvata lugens (Hemiptera: Delphacidae)
Source: J Insect Sci. 2020 Feb 15;20(1):9. doi: 10.1093/jisesa/ieaa002 (PMC7022682; doi:10.1093/jisesa/ieaa002)

## Slide 1
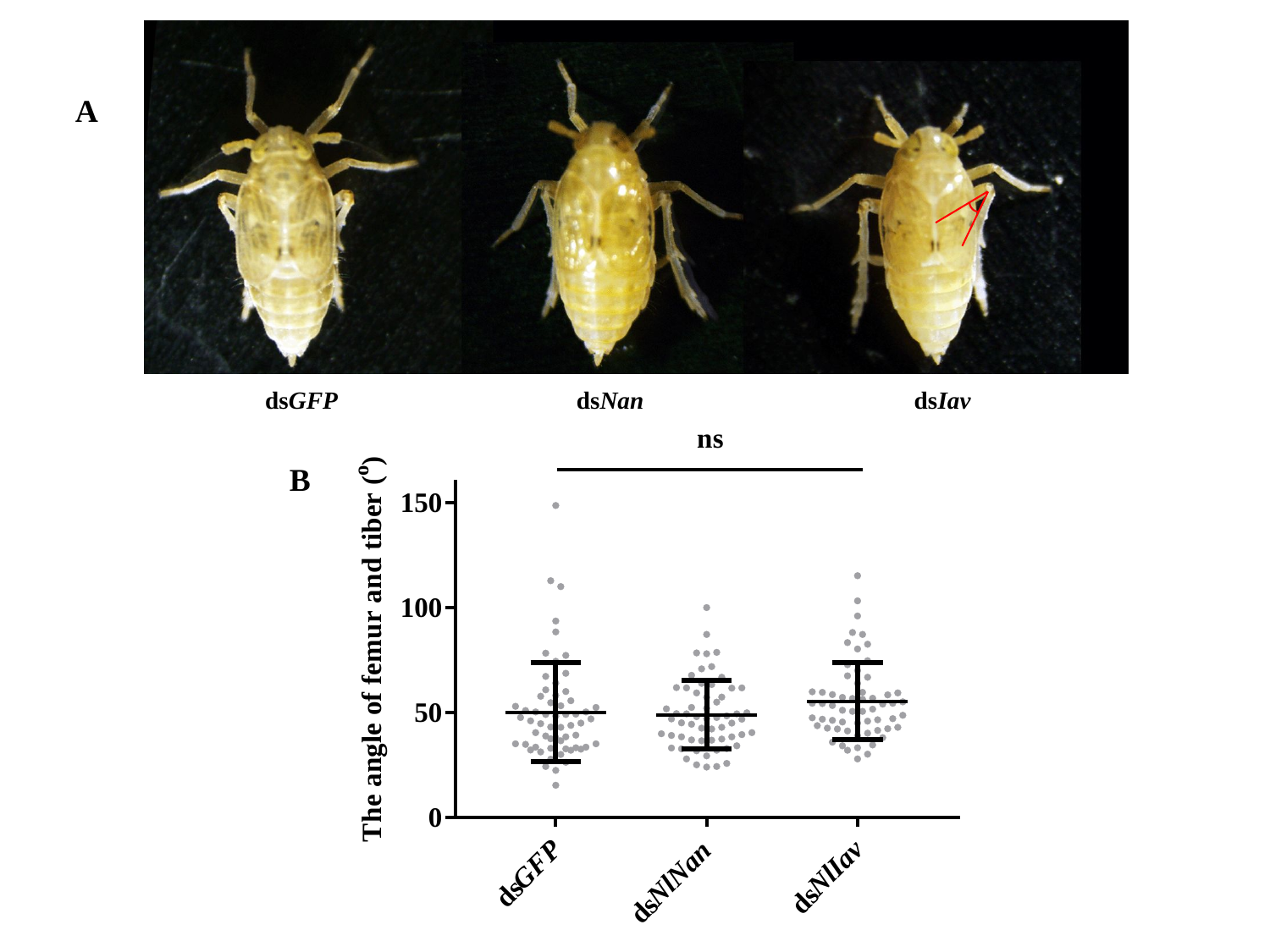

dsGFP dsNan dsIav
A
B

Supplement: ieaa002_suppl_Supplementary_Figure_S1 [file ieaa002_suppl_supplementary_figure_s1.pptx]
